# Rapid Assessment of Trachoma in Underserved Population of Car-Nicobar Island, India

**DOI:** 10.1371/journal.pone.0065918

**Published:** 2013-06-14

**Authors:** Praveen Vashist, Noopur Gupta, Abhilakh S. Rathore, Anita Shah, Suman Singh

**Affiliations:** 1 Department of Community Ophthalmology, Dr. Rajendra Prasad Centre for Ophthalmic Sciences, All India Institute of Medical Sciences, New Delhi, India; 2 Directorate General of Health Services, Ministry of Health & Family Welfare, Government of India, New Delhi, India; 3 Govind Ballabh Pant Hospital, Port Blair, Andaman & Nicobar Islands, India; University of California, San Francisco, University of California, Berkeley, and the Children’s Hospital Oakland Research Institute, United States of America

## Abstract

**Purpose:**

To determine the burden of trachoma and its related risk factors amongst the native population of Car-Nicobar Island in India.

**Methods:**

Rapid assessment for trachoma was conducted in ten villages of Car- Nicobar Island according to standard WHO guidelines. An average of 50 children aged 1–9 years were assessed clinically for signs of active trachoma and facial cleanliness in each village. Additionally, all adults above 15 years of age in these households were examined for evidence of trachomatous trichiasis and corneal opacity. Environmental risk factors contributing to trachoma like limited access to potable water & functional latrine, presence of animal pen and garbage within the Nicobari hut were also noted in all villages.

**Results:**

Out of a total of fifteen villages in Car-Nicobar Island, ten villages were selected for trachoma survey depending on evidence of socio-developmental indicators like poverty and decreased access to water, sanitation and healthcare facilities. The total population of the selected clusters was 7277 in the ten villages. Overall, 251 of 516 children (48.6%;CI: 46.5–55.1) had evidence of follicular stage of trachoma and 11 children (2.1%;CI:1.0–3.4) had evidence of inflammatory stage of trachoma. Nearly 15%(CI:12.1–18.3) children were noted to have unclean faces in the ten villages. Trachomatous trichiasis was noted in 73 adults (1.0%;CI:0.8–1.2). The environmental sanitation was not found to be satisfactory in the surveyed villages mainly due to the co-habitance of Nicobari people with domestic animals like pigs, hens, goats, dogs, cats etc in most (96.4%) of the households.

**Conclusion:**

Active trachoma and trachomatous trichiasis was observed in all the ten villages surveyed, wherein trachoma control measures are needed.

## Introduction

Trachoma, the leading cause of infectious blindness globally, [1] usually affects the most socio-economically disadvantaged regions of the world. According to recent estimates, trachoma is endemic in 57 countries of the world and India is one of the five countries accounting for nearly half of the global burden of active trachoma. [2].

Trachoma related blindness was a major public health problem in India during 1959–63 with active trachoma rates ranging from 56% to 79% in children under 10 years of age in four states of the country. [3] Thereafter, public health interventions and trachoma control measures along with mass antibiotic treatment were undertaken as part of the Trachoma Control Pilot Project by the Government of India with assistance from the World Health Organization (WHO). [4] These activities were then subsumed into the National Programme for Control of Blindness (NPCB), India which was launched in 1976. [5] In 2006, a national survey on trachoma was conducted in the previously endemic states. [6] It was reported that 5.8% of children aged 1–9 years demonstrated clinical signs of active trachoma, while the magnitude of trichiasis was very low (0.15%). [6] Hence it was inferred that trachoma has ceased to be a public health problem in India.

India is committed to eliminate trachoma related blindness by 2020 as partner to the alliance for the Global Elimination of Trachoma (GET) launched by the World Health Organization. [7] In order to achieve elimination of trachoma from all parts of India with a population of nearly 1.21 billion [8], remote, marginalized and underserved populations in the country where trachoma is likely to be endemic need to be surveyed. This survey was undertaken after an alarming number of trachoma cases were reported from Car Nicobar Island by the State Program Officer, Andaman & Nicobar Islands, NPCB, India. A preliminary visit was conducted by a team of experts to confirm whether the reported hospital cases of corneal opacification and blindness could be attributable to trachoma. After confirmation, it was then planned to conduct trachoma rapid assessment (TRA) in this region, in order to generate evidence and prioritize trachoma interventions in the southernmost district of the country.

## Materials and Methods

### Ethics Statement

The study protocol received ethical approval from the State Programme Officer, Directorate of Health Services, Ministry of Health and Family Welfare (MoH&FW), Andaman & Nicobar Islands, Government of India. Clearance to conduct the trachoma survey was also obtained from the District Commissioner and local administrative authorities of Car-Nicobar Islands.

The study was explained to the village chief captains, local health care workers & volunteers, teachers, household heads, and each adult participant. The examination protocol was explained to each adult in their local language. Verbal consent for enrollment and examination was obtained from all adults for their own participation. Consent was documented on the forms by the epidemiologist/field investigator of the respective survey team.

Verbal consent was obtained from parents or appropriate guardians of eligible children before they were included in the study in accordance with the principles of the declaration of Helsinki. Written approval for this protocol and for the consent procedure was obtained from the State Programme Officer, Directorate of Health Services. The study involved basic ocular examination according to standard of care and no invasive procedures were performed on any of the participants during the survey.

Households or individuals for whom informed consent was not given were not included in the survey. Household members that were unwell were not examined for trachoma. Personal identifiers were removed from the dataset before analysis.

### Training for the Survey

A two day workshop was conducted at the Bishop John Hospital, District Hospital of Car-Nicobar Island by the trainers from Community Ophthalmology Department, Dr. Rajendra Prasad Centre for Ophthalmic Sciences, All India Institute of Medical Sciences, New Delhi, India (World Health Organization Collaborative Centre for Prevention of Blindness). The trainers have been involved in conception, design, implementation and co-ordination of previous national surveys and the national trachoma survey in 2006. [6] To confirm reliability of clinical grading of trachoma cases, agreement analysis was done between the two ophthalmologists involved in the current survey. The cases for assessing the agreement were identified from the ophthalmic outpatient department of the Bishop John Hospital, Car-Nicobar Island. The two ophthalmologists were also assessed for their agreement with the standard WHO slides in the final grading examination. A kappa score of more than 0.8 for grading of active trachoma was acceptable between the two graders or each grader with standard WHO slides. Standardization of all field procedures, including trachoma grading, form filling, facial cleanliness status grading, and data entry was ensured at all stages.

### Survey Design & Methodology

The WHO standard methodology for TRA was followed for the present trachoma survey conducted in April 2010.This methodology involves purposive sampling of deliberately choosing those villages/communities where trachoma is likely to be present. [9] Some modifications were incorporated in the standard methodology in order to customize the survey design to Indian conditions. These relate to relatively large sizes of Indian villages, so a segmented village cluster with a population of nearly 500–1000 was selected for TRA. As in previous national trachoma surveys of India, [6] adult males above 15 years of age were also included for trachoma examination along with the females. All clinical protocols were exactly the same as recommended by WHO. TRA is a preferred, low cost methodology for rapid and non random prioritization of high risk communities most likely to have trachoma.

A purposive sampling method was used, as recommended by WHO. [9] To obtain “worse case estimates”, ten out of fifteen villages in Car-Nicobar were prioritized for sampling based on known risk factors for trachoma such as lack of water & basic sanitation facilities, evidence of overcrowding, and poor socio-economic status. The list consisting of ten villages at highest risk of developing trachoma was prepared by the District Program Manager, Blindness Control Programme. In each village, the socio-economically disadvantaged segment of the village (based on socio-economic, environmental, sanitation and water availability indicators) with population of 500–1000 was identified in consultation with the community leaders (village captain). In each selected cluster, nearly 50 children less than 10 years of age were examined for signs of active trachoma through house-to-house visits by the study team. Subsequent households were selected as the next nearest until the desired sample size was reached. All adults over 15 years of age were examined for presence of TT in the same households where children were examined. Besides this, focused group discussions (FGDs) were also conducted in the Community Hall of each village at the commencement of the survey in that village, to obtain community perception on trachoma. Individuals whom the key informants or the participants of focused group discussions could identify as suspected trichiasis were recorded and contacted by the ophthalmologists for confirmation as a case of TT.

### Ophthalmic Examination & Risk Factor Assessment

All participants were examined for signs of trachoma with a flash light and a 2.5× binocular loupe. The findings were graded according to the standard WHO simplified grading system. [10] ‘Active trachoma’ was defined as trachomatous follicular inflammation (TF) and/or trachomatous inflammation intense (TI) in either eye. ‘Scarring trachoma’ included trachomatous scarring (TS), trachomatous trichiasis (TT) and/or corneal opacity (CO) in either eye. TT was defined as at least one eyelash rubbing the eyeball or evidence of recent removal of in-turned eyelashes. Observation of facial hygiene was done in all the children examined for active trachoma; wherein unclean faces were defined as either presence of discharge from the eyes/nose, crusting of discharge around the eye or nose, or presence of flies around eye/nose.

The survey team including the ophthalmologist, optometrist, local health worker and the epidemiologist inspected the general condition of each village, with special emphasis on the living conditions and environmental factors like distance of water source from the household, presence of functional latrine and presence of solid waste/animal pens within the household premises. Access to health care and general facilities like primary health care centre, village pharmacy, market and school was also assessed for 25–30 households per village.

### Data Management & Statistical Analysis

Information on the questionnaires was double entered into an electronic Microsoft Office Excel ® version 2007 database at a central location, cleaned and the analysis was completed using SPSS (version 14.0, SPSS, Inc., Chicago, USA) and Stata (version 10.0, Stata Corp., College Station, TX, USA). Frequency distributions of study participants were explored and chi-square tests were used to determine association between occurrence of disease and associated risk factors. Cross tabulations were used to describe the frequency distribution of characteristic among the sample population. Potential risk factors were categorized into sanitary (e.g., facial cleanliness, presence of fly-eye), demographic (e.g., gender, age) and environmental factors (e.g., accessibility to water and functional latrine, presence of animal pens) and analyzed accordingly. Any p value less than 0.05 was considered statistically significant.

## Results

**Figure 1:**
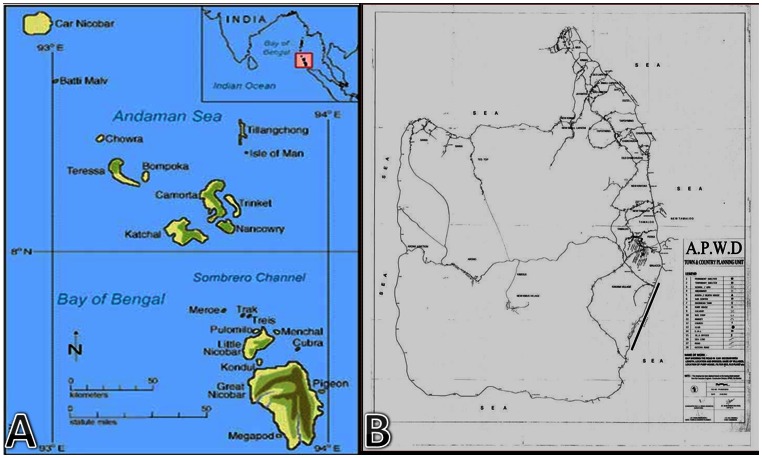
Location of the survey area. A) Map of Southern India (upper right inlet) showing location of Andaman & Nicobar Islands. Nicobar is the southernmost district of the Indian subcontinent. B) Detailed map of Car-Nicobar Island.

The island of Car-Nicobar, district of Nicobar, Andaman & Nicobar Islands, India (Fig. 1) comprises of 15 villages with a total population of 17,967. Ten villages (population = 11,334) were included in the Rapid Assessment. The total population of the ten selected clusters where the TRA was conducted was 7277. A total of 516 children in the island were examined for clinical signs of active infection due to trachoma. The number of children examined per village ranged from 48–54, average being 52. In accordance to TRA guidelines, the number of pre-school children included in the study (aged one to four years) was nearly equal to those examined in the age group of five to nine years (1∶1.2). The total number of adults examined for evidence of trichiasis and corneal opacity attributable to trachoma was 970. Males contributed to nearly 40% of the adult population included in the study.

### Active Trachoma in Car-Nicobar

Two hundred and sixty two children (50.8%;CI:46.6–55.3) demonstrated signs of active trachoma infection with the proportion of infected children ranging from 37.5% to 73.0% in the ten villages. Among them, follicular stage of trachoma (TF) was noted in 251 children (95.8%) while 11 children (4.2%) demonstrated inflammatory changes (TI) due to active trachoma infection. Unclean faces were seen in 77 children (14.9%;CI:12.1–18.3) of Car-Nicobar Island with a wide variation (1.9% to 56.6%) amongst the ten villages (Table1).

**Table 1:** Distribution of children with signs of active trachoma infection & its related risk factors in Car Nicobar Island.

Village name	Populationof Cluster	Childrenexamined	Children withTF/TI n (%)	UncleanFaces n(%)	No. ofHouseholdsassessed	Presence of SolidWaste or AnimalPens	Absence ofFunctional Latrine
**KINYUKA**	603	52	38(73.1)	8(15.3)	30	28(93.3)	1(3.3)
**CHUKCHUCHA**	580	54	24(44.4)	3(5.6)	23	22(95.7)	0
**ARONG**	600	50	23(46.0)	8(16.0)	26	25(96.2)	0
**TAMALOO**	700	52	23(44.2)	4(7.7)	30	30(100.0)	2(6.6)
**KAKANA**	896	53	24(45.3)	3(5.7)	29	29(100.0)	0
**BIG LAPATHY**	700	50	29(58.0)	6(12.0)	29	29(100.0)	0
**TAPOIMING**	925	52	27(51.9)	8(15.4)	24	24(100.0)	0
**SMALL LAPATHY**	940	52	31(59.6)	1(1.9)	30	27(90.0)	0
**MUS**	700	48	18(37.5)	6(12.5)	25	25(100.0)	1(4.0)
**KINMAI**	633	53	25(47.2)	30(56.6)	30	27(90.0)	1(3.3)
**TOTAL**	7277	**516**	**262(50.8)**	**77(14.9)**	**276**	**266(96.4)**	**5(1.8)**

TF = Trachoma Follicular; TI = Inflammatory stage of trachoma.

### Trichiasis Load in Car-Nicobar

Nicobari tribes in all the ten villages demonstrated evidence of TT. Seventy three cases of trichiasis were detected by house-to-house visits for clinical examination and also when reported by FGDs/key informants & confirmed by clinical examination in a population cluster of 7277 in 10 villages, amounting to a trichiasis load of 1% (CI:0.8–1.2). Corneal opacity was present in 30% of cases with TT (Table 2). The proportion of cases with severe entropion amounted to 15% of TT cases. Eight percent of the patients with TT demonstrated features of recurrent entropion due to trachoma.

**Table 2:** Burden of Trachomatous Trichiasis in adults residing in Car Nicobar Island.

Village name	Population of Cluster	TT Cases	TT Without CO	TT With CO	Burden of TT in Community*
**KINYUKA**	603	11	5	6	18.2
**CHUKCHUCHA**	580	6	5	1	10.3
**ARONG**	600	1	1	0	1.7
**TAMALOO**	700	15	7	8	21.4
**KAKANA**	896	5	5	0	5.6
**BIG LAPATHY**	700	7	3	4	10.0
**TAPOIMING**	925	11	4	7	11.9
**SMALL LAPATHY**	940	2	0	2	2.1
**MUS**	700	11	11	0	15.7
**KINMAI**	633	4	4	0	6.3
**TOTAL**	7277	73	45	28	10.0

* Per 1000 population.

TT = Trachomatous Trichiasis; CO = Corneal Opacity.

### Environmental Indicators

In Car-Nicobar Island, 97.5% of residents had access to potable water within 30 minutes of walking distance from their households. Functional latrine was available in most of the households (98.2%). In contrast, the environmental sanitation was not found to be satisfactory mainly due to the co-habitance of people with animals like pigs, hens, goats, dogs, cats etc. in Nicobar. Most of the households in Nicobar (96.4%) had an animal pen within the household premises or at the base of the Nicobari hut/dwelling. Presence of animal pens in close vicinity to the households was significantly associated (p = 0.04) with occurrence of TF in children aged 1–9 years. There is an absence of proper water drainage system in all the ten villages surveyed. It was noted that there is no proper garbage disposal facility available anywhere in the island.

### Access to Facilities

Car-Nicobar does not have access to a trichiasis treatment facility. The patient needs to leave the island and travel through helicopter or ship to Port Blair, capital city of Andaman & Nicobar Islands, for availing surgical services. The access to primary health centre and village pharmacy (sub-centre) was better. All the villages had access to a subcentre and basic health services were being provided by ANM (Auxiliary Nurse Midwife) at these subcentres. ASHA (Accrediated Social Health Activists) workers have been appointed under National Rural Health Mission, Government of India in all the villages. Primary schools were within walking distance, but market facilities were farther than 30 minutes of walking distance in four villages.

## Discussion

The trachoma survey at Car-Nicobar was the first population based survey on trachoma in this remote and underserved ‘tribal reserve’ area. Geographical remoteness and inaccessibility of this region from the mainland means that it is often difficult and costly to conduct fieldwork in these underserved areas; consequently there is limited data on the distribution of trachoma in such regions of the world. The present survey was conducted in accordance with the WHO guidelines for TRA and demonstrated a very high active trachoma infection rate (TF/TI) of 50.8% among children in 1–9 year age group and nearly one percent of the population afflicted with trachomatous trichiasis. A possible limitation of the present study was non-availability of microbiological investigations and their results to correlate with the clinical findings of active trachoma in these children. SAFE strategy measures like surgical facilities for patients with TT, mass azithromycin treatment, health education for facial cleanliness and hand washing and measures to improve water and sanitation should be undertaken to eliminate blinding trachoma in this underserved island.

Co-habitance of Nicobari people with animals like pigs, hens, goats, dogs, cats etc. could be a contributory risk factor for high occurrence of trachoma in this population. Pets were observed in close vicinity of most of the households. Insufficient environmental sanitation, particularly for sewage and garbage disposal at the community level and, keeping cattle and animals next to human dwellings make fly breeding possible close to households, thus facilitating transmission of trachoma infection. As exemplified in published literature, personal and environmental hygiene are vital determinants for occurrence and spread of trachoma. [11] Accumulated animal excreta and animal manure are an important source of fly breeding. [11] Although human faeces may be the preferred larval medium for the housefly, young *Musca sorbens* have been reported emerging from pig, dog, milk-fed calf and cattle faeces in addition to that of humans. Removal of human faeces from the environment, through improved sanitation facilities is likely to reduce trachoma transmission, but if faeces of other animals are present, *M*. sorbens will persist. [12]Many studies have shown that the presence of animals and animal dung within the households is an independent risk factor for occurrence of active trachoma. [13], [14].

It was noted that the living standards and socio-environmental factors were similar in most of the surveyed clusters and within the clusters with not much variation in Car Nicobar Island. The effectiveness of environmental sanitary measures on the prevalence of active trachoma in endemic areas has been studied extensively. [13] Improvement of personal and community hygiene has great potential for a sustainable reduction in trachoma transmission. [15] Environmental improvement includes increasing access to water, use of latrines and other fly control interventions, moving animals away from the household environment; education, both general and specific for trachoma; and improved local economy leading to better living conditions. [16] Environmental factors like climate and altitude have also been linked to trachoma. Warm and dry climate zones have reported higher trachoma prevalence rates [17], [18] while at high altitude, prevalence of trachoma is lower. [19]In addition, common risk factors for active trachoma reported by trachoma surveys conducted globally include dirty face, [20] nasal and ocular discharge, [21] flies on the face and overcrowding. [22].

The district administration at Car-Nicobar should use effective health promotion tools for educating people to improve hygiene so as to decrease transmission of trachoma. The people of Car-Nicobar should be educated about trachoma and its spread, encouraging acceptance for surgery and antibiotic treatment, encouraging facial cleanliness and promoting clean environment. There is a need for promoting interpersonal communication and warn people for behavioral changes and preference for nuclear families. The most appropriate channel for health promotion is community meetings in the villages where group discussions with the villagers can be used to convey information about trachoma and its control. The ANMs and ASHA workers, who act as key local health volunteers may be given training to discuss important topics like causes of spread of trachoma, trachoma surgery, antibiotic treatment, facial cleanliness, hand washing and environmental changes in the community meetings.

The results of the survey demonstrated high occurrence of trichiasis in this remote island of the country. Trachomatous trichiasis including some cases of blinding trachoma was evident in 73 adults. Blindness due to trachoma is irreversible once it has occurred, but it can be avoided. Provision of timely intervention and surgery at the earliest, is warranted to prevent development of corneal blindness due to trachoma. There is a need to strengthen the district hospital to provide adequate facilities for surgical correction of entropion and trichiasis. The results also highlight the importance of key informants and FGDs, and in this study their information led to trachomatous trichiasis being identified in all the villages of the island.

The results of the two national trachoma surveys in India conducted in 2006–07 and 2010 have been summarized in Table 3. It is evident that the magnitude of active trachoma and trachomatous trichiasis in the ten districts of six previously hyper-endemic states of India, has markedly decreased to a level that it is no more a public health problem in the country. As highlighted previously, Car Nicobar Island needs to be treated as a priority area for trachoma intervention and control programmes.

**Table 3:** Comparative data on magnitude of active trachoma and trachomatous trichiasis in districts covered during 2006 and 2010 NPCB, India trachoma rapid assessment survey.

Districts	Population ofexamined cluster	No. ofTT cases	Magnitudeof TT (%)	Number ofchildrenexamined	Number ofchildren withTF/TI	Proportion ofChildren withsigns of ActiveTrachoma (%)
**Kutch** *	7700	2	0.03	530	3	0.6
**Banaskantha** *	9190	4	0.04	525	6	1.1
**Dholpur** *	7050	22	0.31	520	33	6.3
**Tonk** *	6370	2	0.03	515	26	5.0
**Bikaner** *	6520	4	0.1	536	61	11.4
**Bullandshahr** *	6300	17	0.3	629	37	5.9
**Pauri Garhwal** *	4015	2	0.05	462	70	15.2
**Mahendergarh** *	7705	5	0.1	500	11	2.2
**Mewat** *	5995	31	0.5	499	29	5.8
**Hoshiarpur** *	4950	11	0.2	548	31	5.5
**Car Nicobar** #	7277	73	1.0	516	262	50.8

* Ten districts of six previously hyper-endemic states of India covered during 2006–07 survey.

# Car Nicobar district, Andaman & Nicobar Islands, India covered during 2010 survey.

TT = Trachomatous trichiasis; TF = Trachoma Follicular; TI = Inflammatory stage of trachoma.

In conclusion, although trachoma may be in the elimination phase in major part of India, it may be present in remote, inaccessible and focal pockets where the need to reach out and implement SAFE strategy and trachoma interventions may be greatest. This survey demonstrates that remote areas of the country should be surveyed and active surveillance and reporting of trachoma cases should be undertaken as a continuous process in order to achieve elimination of trachoma from India by 2020.
